# Lymphoepithelioma-like carcinoma with carcinosarcoma of the endometrium: a case report and literature review

**DOI:** 10.3389/fimmu.2025.1636575

**Published:** 2025-07-21

**Authors:** Jialing Xu, Bin Huang, Yanhua Zhu, Jianping Shi, Qiang Zhou

**Affiliations:** ^1^ Departments of Medical Oncology, The First People’s Hospital of Xiaoshan District, Hangzhou, Zhejiang, China; ^2^ Department of Pathology, The First People’s Hospital of Xiaoshan District, Hangzhou, Zhejiang, China; ^3^ Outpatient infusion Room, Chengxiang Health Service Center, Xiaoshan District, Hangzhou, China; ^4^ Department of Anorectal Surgery, The First People’s Hospital of Xiaoshan District, Hangzhou, Zhejiang, China

**Keywords:** carcinosarcoma, clinicopathology, endometrium, lymphoepithelioma-like carcinoma, molecular pathology

## Abstract

This report describes a 56-year-old female diagnosed with lymphoepithelioma-like carcinoma with carcinosarcoma of the endometrium (LELCCSE). She experienced vaginal bleeding and lower abdominal discomfort without any obvious cause following six years of menopause. She sought treatment at our hospital in Hangzhou, China. An ultrasound examination revealed a hypoechoic mass within the uterus. Subsequently, a biopsy confirmed it as lymphoepithelioma-like carcinoma (LELC) with carcinosarcoma.The patient underwent a total hysterectomy, bilateral salpingo-oophorectomy, and associated lymphadenectomy. The postoperative pathology suggested LELC complicated with carcinosarcoma and an IIC grade by the 2023 FIGO staging system. She received adjuvant chemotherapy and radiotherapy without any molecular testing due to her financial constraints. A gene which encodes the catalytic subunit of DNA polymerase epsilon(POLE) mutation was found by the retrospective molecular testing; however, no recurrence or metastasis occurred after 12 follow-up months.

## 1 Introduction

Lymphoepithelial carcinoma is an undifferentiated carcinoma that was initially identified in the nasopharynx. Associated with the Epstein-Barr virus (EBV), it is characterized by undifferentiated epithelial tumor cell clusters and significant lymphocytic as well as plasma cell infiltration (1–3). Cancers with similar morphological features and occurring outside the nasopharynx are called “lymphoepithelioma-like carcinoma” (LELC). Although they have been reported in several anatomical sites (4–6), their occurrences in the endometrium are rare (7–13). The main clinical symptom of endometrial LELC is vaginal bleeding post-menopause, similar to the symptoms of other common gynecological conditions. The diagnosis of endometrial LELC relies on pathological assessment, immunohistochemistry, and molecular pathology analysis. Moreover, immunohistochemical markers like Epithelial Membrane Antigen(EMA) and Cytokeratin(CK) show positive expressions, while molecular Small RNA encoded by Epstein-Barr virus(EBER) displays a negative result. Additionally, endometrial carcinosarcoma is complex and highly malignant (14–16). Because of variable biological behaviors and prognostic characteristics, its existence with endometrial LELC makes both diagnosis and treatment difficult. Recent molecular pathology advancements have promoted the relevance of gene mutation analysis for classifying and assessing the prognosis of endometrial cancer. Notably, the POLE gene’s mutation status is significantly associated with a better prognosis for patients (17, 18). We have described a 56-year-old female patient diagnosed with POLE-mutated Lymphoepithelioma-like Carcinoma with Carcinosarcoma of the Endometrium (LELCCSE). This rare case highlights the necessity of molecular pathology and offers new perspectives for timely and appropriate diagnosis as well as treatment in clinical practice.

## 2 Case report

A 56-year-old female who had been postmenopausal for six years reported a single episode of minimal bright red vaginal bleeding and mild lower abdominal pain six months ago. However, she did not seek immediate medical attention and was admitted to our hospital in Hangzhou, China, 11 days ago. A physical examination indicated a normal general condition. Moreover, the gynecological assessment showed no abnormalities in the vulva, vagina, or cervix. The uterus was in a normal position, firm, without any palpable masses, and also showed moderate mobility without any tenderness. No significant masses were observed in the adnexal regions.

**Figure 1:**
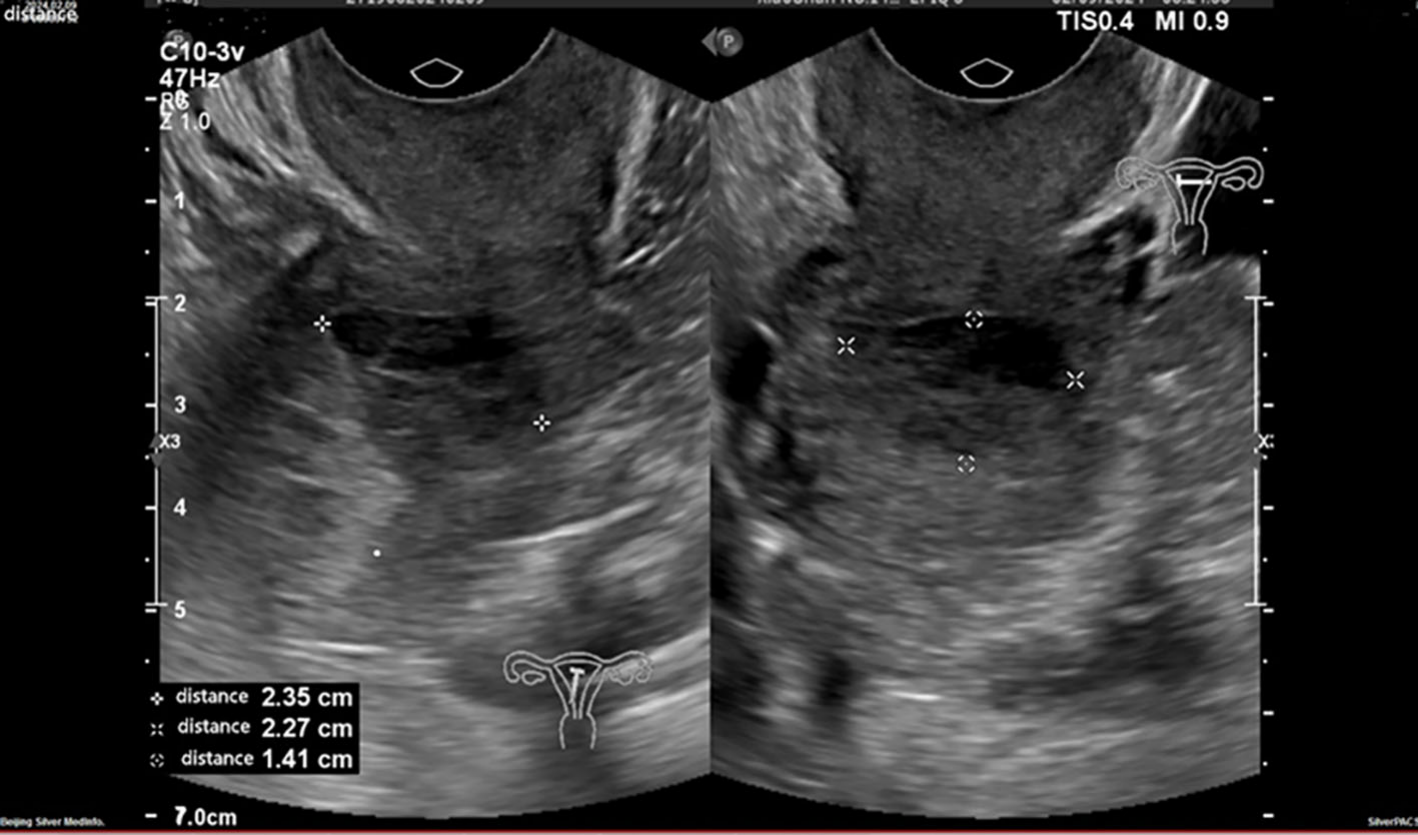
An ultrasound examination reveals a hypoechoic mass in the left posterior wall of the uterus.

An ultrasound revealed that the uterus was in a normal position. The uterine cavity was completely compressed by a hypoechoic mass with clear borders, measuring approximately 2.4 x 2.3 x 1.4 on the left uterine body’s posterior wall (**Figure 1**). Its endometrial thickness (single layer) was 0.7 cm. Heterogeneous echogenicity and blood flow signals were also observed. In the left ovary, a cystic dark area, measuring approximately 3.0 x 2.7 x 2.4 cm, contained clear internal fluid. The right ovary was faintly visible, smaller, and displayed a relatively solid echogenicity. A pelvic Magnetic Resonance Imaging (MRI) scan with enhancement was performed, and revealed a soft tissue mass measuring approximately 3.0 x 2.0 cm within the uterine cavity. The serum tumor markers were: alpha-fetoprotein at 1.39 ng/mL (normal range: 0.89-8.78 ng/mL), carcinoembryonic antigen at 2.58 ng/mL (normal range: 0.00-5.00 ng/mL), and carbohydrate antigen 125 at 12.70 U/mL (normal range: 0.00-35.00 U/mL).

**Figure 2:**
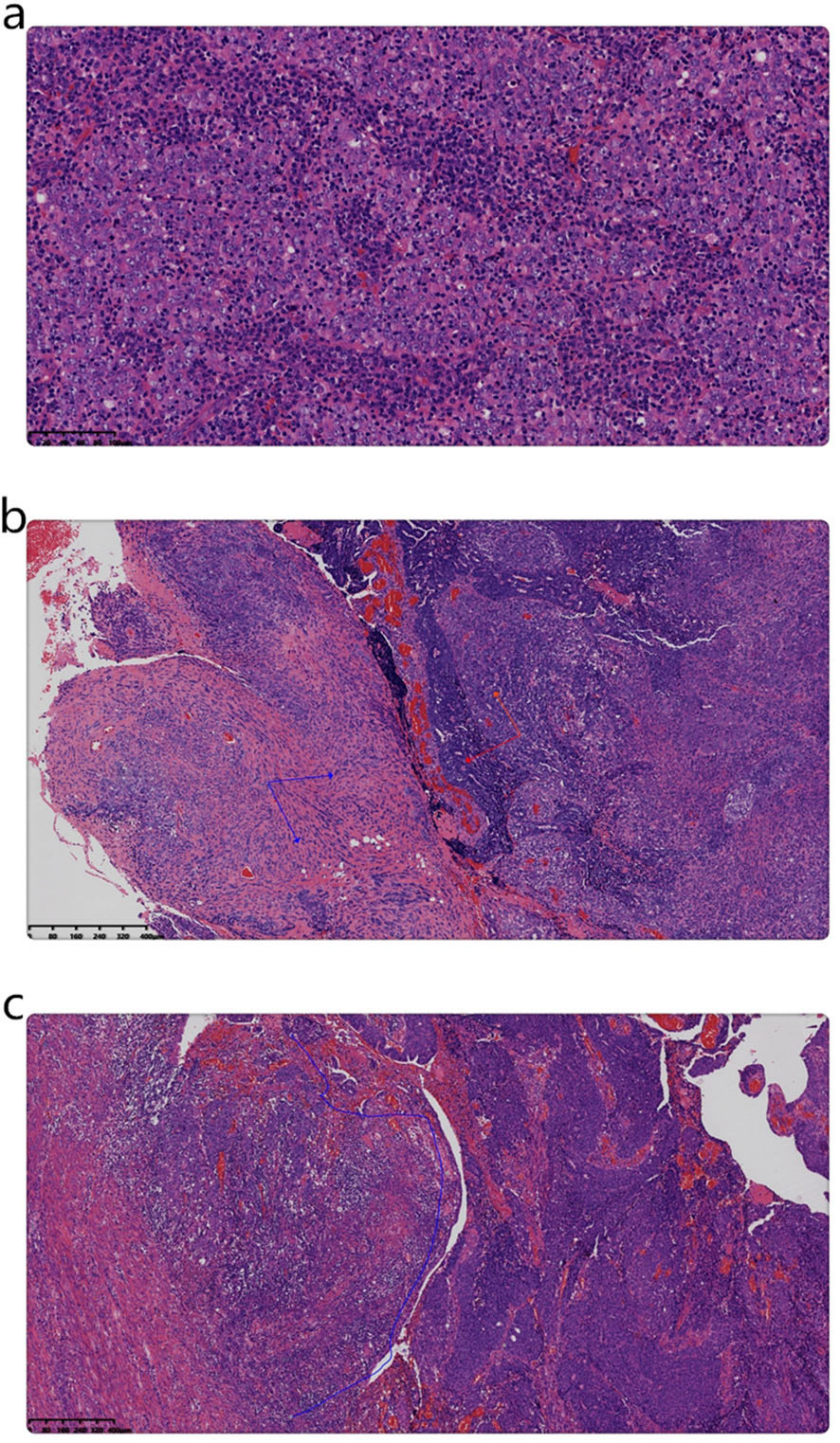
**(a)** Microscopic examination of the biopsy indicates that the tumor was composed of nests of undifferentiated carcinoma cells, including syncytial cells and vacuolated nuclei, along with abundant lymphocytic and plasma cell infiltration. (Magnification, x200; the scale bar measured 100µm; H&E). **(b)** Microscopic examination of the surgical specimen shows that the tumor comprised poorly differentiated endometrioid carcinoma (red arrow) and intermediate-grade sarcoma (blue arrow) components. (Magnification, x50; the scale bar measured 400µm; H&E). **(c)** At the base of the mass, poorly differentiated endometrioid carcinoma (right-side blue line area) was observed adjacent to a smaller LELC component (left area of the blue line). (Magnification, x50; the scale bar measured 400µm; H&E).

Married at 21 years, the patient had four pregnancies: four full-term, none preterm, two abortions, and four living children. Her endometrial biopsy showed a fragmented dark red tissue, measuring 3x2x1 cm. Microscopic examination revealed the tumor comprised nests of undifferentiated carcinoma cells with prominent and vacuolated nuclei, a small carcinosarcoma component, and extensive lymphocytic and plasma cell infiltration (**Figure 2a**).

Subsequently, the patient underwent a laparoscopic transabdominal total hysterectomy, bilateral ovariectomy, salpingectomy, pelvic lymph node dissection, and para-aortic lymph node resection. The gross examination revealed an intact uterine specimen. The uterine body volume measured 6x5x3 cm. Although the endometrium was thin, the muscle wall thickness ranged from 1-2 cm. A polypoid mass measuring 3 x 2 x 2 cm was found at the uterine cavity’s base. The cut section was gray with a medium texture and displayed superficial muscle infiltration. The cervix measured 3x2.5x2 cm and exhibited a smooth surface.

The gynecologic specimen’s microscopic examination revealed that the tumor comprised a mixture of poorly differentiated endometrioid carcinoma and intermediate-grade spindle cell sarcoma. Additionally, a small area of LELC was observed adjacent to the tumor base (**Figures 2b, c**). It infiltrated the superficial myometrium (infiltration 0.5/total thickness 1.5cm) without any evidence of vascular invasion.

**Figure 3:**
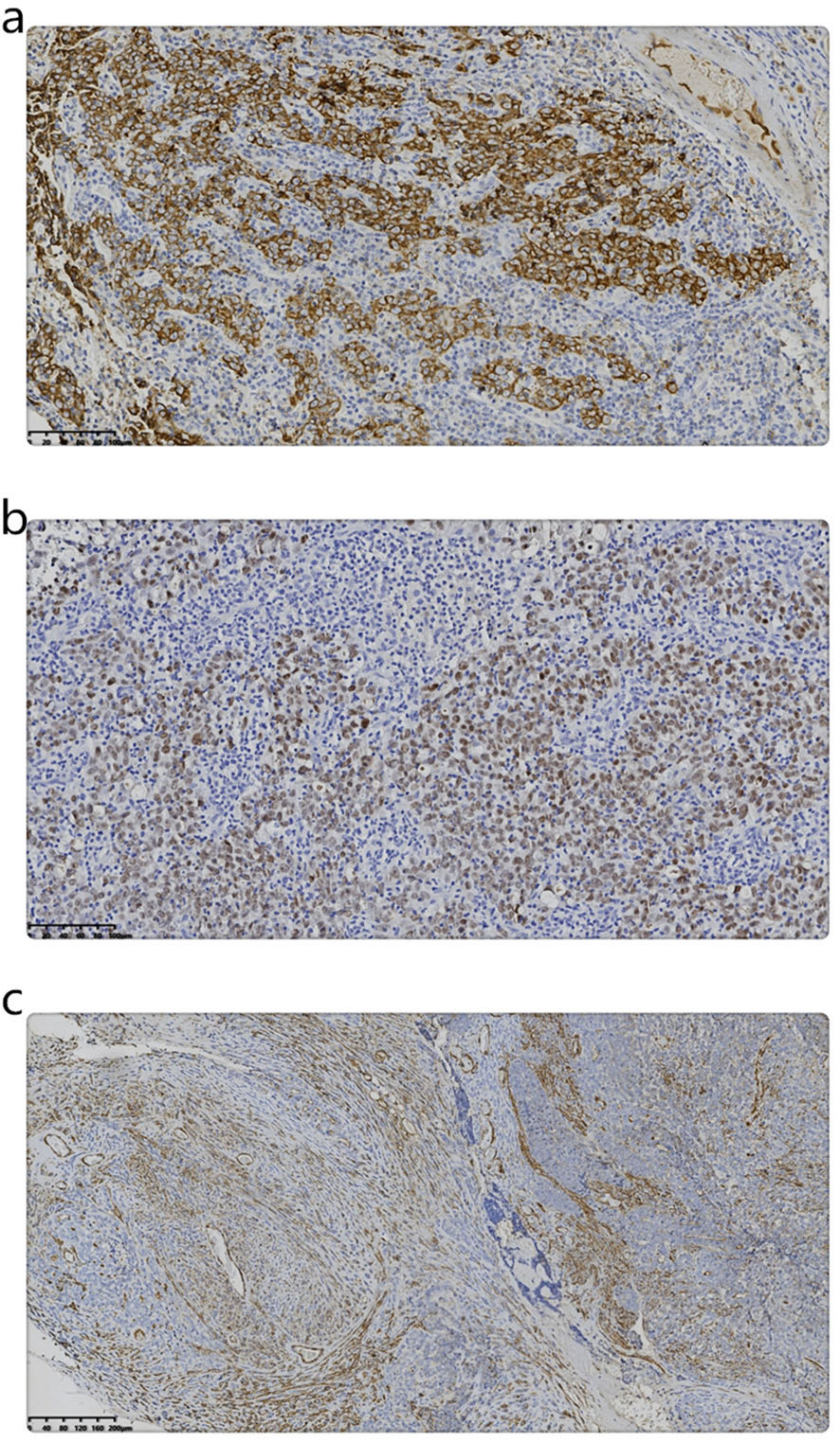
**(a)** Immunohistochemistry shows the expression of EMA in LELC. (Magnification, IHC x200; the scale bar measured 100 µm). **(b)** Immunohistochemistry shows the expression of Pax8 in LELC. (Magnification, IHC x200; the scale bar measured 100 µm). **(c)** Immunohistochemistry shows the expression of SMA in sarcoma. (Magnification, IHC x100; the scale bar measured 200 µm).

**Table 1** displays the results of immunohistochemical and molecular pathology assessments. The LELC expressed both EMA and Pax8 (**Figures 3a, b**). Conversely, a part of the sarcoma expressed smooth muscle actin (SMA) also (**Figure 3c**). However, *in situ* hybridization evaluation was negative for EBER.

**Table 1:** Immunohistochemical and molecular detection results of LELCCSE.

Tumor name		ER	PR	Pax8	P16	SMA	P53	P63	Vim	CK7	CK5/6	EMA	CyclinD1	MSH2	MLH1	PMS2	MSH6	EBER
LELC		N	N	P	PP	N	10%P	N	N	N	N	P	P	P	P	P	P	N
CS	EC	PP	PP	P	P	N	80%P	PP	PP	P	PP	P	N	P	P	P	P	N
	SC	N	N	N	P	PP	20%P	N	P	N	N	N	N	P	P	P	P	N

EC, Endometrioid carcinoma; SC, sarcoma; N, Negative; PP, Partly Positive.

The pathological diagnosis was lymphoepithelioma-like Carcinoma with Carcinosarcoma of the Endometrium (LELCCSE). Infiltrating the superficial myometrium(<1/2), it did not involve the cervix. Bilateral ovaries and fallopian tubes exhibited serous cystadenofibromas and salpingitis, respectively. Bilateral pelvic lymph nodes showed negative results in ten nodes, and para-aortic lymph nodes were not detected. Thus, it was graded as IIC by the 2023 FIGO staging system.

Treatment: After gynecologic surgery, the patient went to another hospital as she declined molecular testing. She underwent four intravenous carboplatin and paclitaxel chemotherapy cycles, four uterine afterloading treatments, and two additional intravenous carboplatin and paclitaxel chemotherapy cycles. Due to myelosuppression, the carboplatin dose was adjusted between 550-680 mg/dose, and the paclitaxel dosage was maintained at 260 mg/dose.

Retrospective molecular testing: A complementary, independent endometrial cancer-related gene sequencing identified LELC and carcinosarcoma tissues (Jinan Kingmed Medical Laboratory Center Co. Ltd). Using the GRCh37/hg19 reference genome, the sequencing was performed using next-generation sequencing (NGS) on the Illumina platform (NextSeq 550/NovaSeq 6000). The samples primarily included single-nucleotide variants (SNVs), small fragment deletions and insertions (INDELs), and copy number variations (CNVs). These findings support the molecular classification and prognostic evaluation of endometrial cancer, as well as predict responses to targeted therapies and immunotherapy.

**Figure 4:**
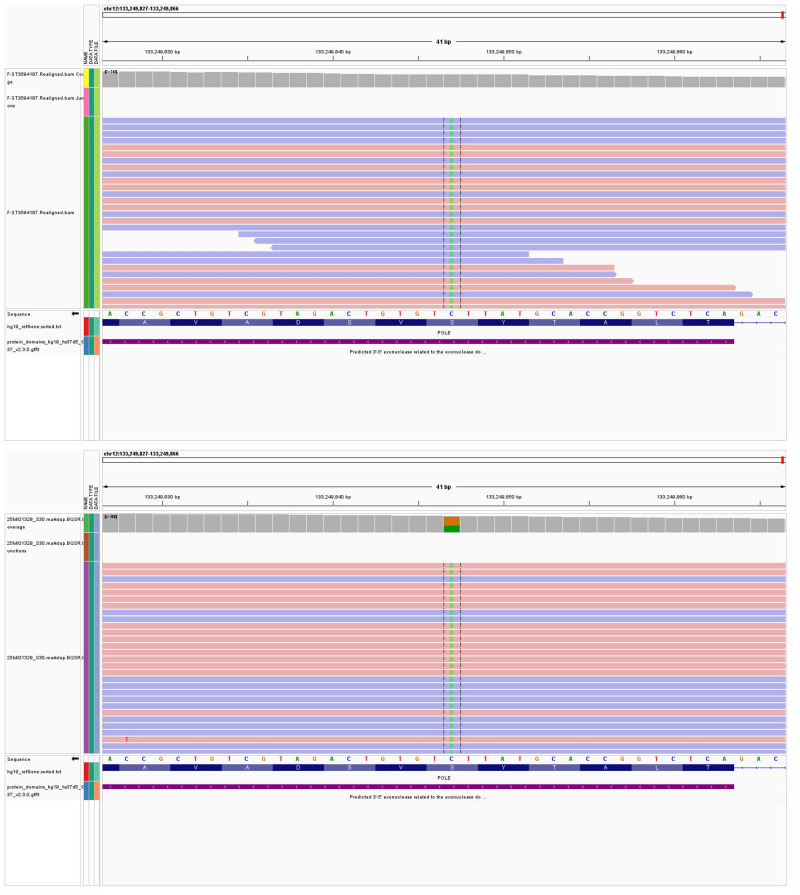
**(A)** POLE mutations (c.1376C>T p.S459F) occurring at frequencies of 18.53% in LELC. **(B)** POLE mutations (c.1376C>T p.S459F) occurring at frequencies of 42.8% in carcinosarcoma.

Our SNV/INDEL results revealed POLE mutations (c.1376C>T p.S459F) occurring at frequencies of 18.53% and 42.8% in LELC and carcinosarcoma (**Figure 4**), respectively. These mutations were classified as Level I, thereby indicating a favorable prognosis and a good response to immunotherapy. Since TP53 mutations (c.637C>T p.R213*) were found at frequencies of 7.86% and 42.1%, they were also classified as Level I. Moreover, TP53 mutations (c.844C>T p.R282W) were identified at frequencies of 4.84% and 41.2%, and classified as Level I. Microsatellite instability was absent.

Finally, the pathological results were updated. Endometrial lymphoepithelioma-like carcinoma with carcinosarcoma was classified as POLE mut and IAm-POLE mut by the WHO molecular classification and 2003 FIGO staging system, respectively.

Follow-up: There was no recurrence or metastasis post-12 follow-up months.

## 3 Discussion

We have presented a rare case of LELCCSE to highlight its important morphological, immunohistochemical, and molecular pathology characteristics. Endometrial lymphoepithelioma-like carcinoma is a rare instance, and only nine cases have been reported together, including this case to date (7–13). Although the primary clinical presentation was postmenopausal bleeding, eight cases were EBV-negative, and only one case showed EBV positivity in a few tumor cells (11). A total of four tumors, including this case, were assessed as microsatellite stable (10, 12, 13) (**Table 2**). The diagnosis depends on pathological examination, and its clinical symptoms are similar to those of other gynecological diseases. Therefore, this case might provide valuable insights for related studies. Molecular testing in this case also revealed a Pole mutation. The pathogenesis of endometrial lymphoepithelioma-like carcinoma is not clearly related to EBV, but TP53 gene mutation and PIK3CA gene mutation have been found (12, 13), which are speculated to be related to these two gene mutations. We found a Pole mutation and added a new reference.

**Table 2:** Clinical pathology of endometrial lymphoepithelioma-like carcinoma.

Reference no.	Age(year)	Symptoms	Histology	EBV	Radical gynecological surgery, chemotherapy and radiotherapy	Molecular testing	FIGO stage	Follow-up (M)/ survival
1 (7)	74	VBM	LELC	N1	Y/Y/N2	N2	IVB	9/Y
	55	VBM	LELC	N1	Y/Y/Y	N2	IIIC	12/N
2 (8)	57	VBM	LELC	N1	Y/N2/N2	N2	IB	24/Y
3 (9)	67	VBM	LELC	N1	Y/N2/N2	N2	IB	8/Y
4 (10)	79	VBM	LELC+Focal clear cells	N1	Y/N2/Y	MSS	IB	4.5/Y
5 (11)	63	VBM	LELC	A Few cells positive	Y/Y/Y	N2	IIB	12/Y
6 (12)	63	VBM	LELC	N1	Y/N2/Y	TP53、PIK3CAmut、MSS	IA	6/Y
7 (13)	67	VBM	LELC	N1	Y/N2/N2	TP53、PIK3CAmut、MSS	IA	16/Y
present	56	VBM	LELC+CS	N1	Y/Y/Y	POLE、TP53、PIK3CAmut、MSS	IAmPoleMut	12/Y

VBM, Vaginal bleeding after menopause; N1, Negative; N2, No; Y, Yes; MSS, microsatellite stability.

The molecular classification of endometrial cancer significantly affects a patient’s prognosis, especially in POLE mutation cases, which are typically associated with more favorable outcomes (17, 18). The “ultra-mutated” nature of POLE-mutant endometrial cancer displays a high mutational burden and improves responses to immunotherapy (19). Additionally, TP53 mutations are frequently correlated with tumor aggressiveness, highlighting the importance of the interaction between these genetic mutations in patient prognosis. According to the WHO molecular classification for endometrial cancer, the cases with concurrent POLE and TP53 mutations should be classified as POLE mutant type (14, 20). In the present case, the identified POLE mutation (p.S459F) and TP53 mutations (p.R213* and p.R282W) offered valuable insights into the tumor’s biological behavior and helped categorize this patient as a POLE mutant type. Additionally, the IIC stage was changed to IAm-POLE mut in the 2023 FIGO staging system.

Furthermore, the pathological examination revealed that endometrial LELC is morphologically similar to undifferentiated carcinoma of the nasopharynx (7–13). Microscopically, there were clusters of undifferentiated epithelial tumor cells and prominent infiltration of lymphocytes and plasma cells, with vacuolated nuclei and prominent nucleoli. Uterine carcinosarcomas comprise both epithelial and mesenchymal components, showcasing variable morphologies. Epithelial carcinomas might present as endometrioid, serous, or clear cell carcinomas (14, 15). The sarcomatous component might include homologous subtypes, like leiomyosarcoma, endometrial stromal sarcoma, fibrosarcoma, and undifferentiated sarcoma, or heterologous subtypes, including rhabdomyosarcoma, chondrosarcoma, osteosarcoma, and liposarcoma (16). Although LELC shows immunohistochemical CK and EMA expressions, *in situ* hybridization often results in negative EBER findings. Thus, our pathological, immunohistochemical, and molecular findings support the diagnosis of LELCCSE. However, the diversity of tumor components might affect the patient’s treatment options and prognosis.

Endometrial carcinosarcoma is highly malignant, and its treatment includes surgery, radiotherapy, as well as chemotherapy (14, 16). However, endometrial cancer patients with POLE mutations get better clinical results after appropriate treatment, which can guide clinical practice (21). With the coexistence of POLE and TP53 mutations, the POLE mutation might still dominate its biological behavior. Bogani G. et al. reported that 20 endometrial cancer patients (64.5%) with both POLE and TP53 mutations displayed a good prognosis (22). However, this patient refused molecular testing due to monetary reasons and sought chemotherapy and radiotherapy in other hospitals, according to the diagnosis of high-grade endometrial cancer. The 12-month follow-up showed no recurrence or metastasis. POLE mutations were identified by retrospective and complementary molecular testing. Due to the limited number of LELCCSE patients with POLE mutations, more cases are required to improve treatment options and prognosis.

This report had a few limitations. Since our patient did not undergo timely molecular testing due to financial reasons, she underwent radiotherapy and chemotherapy for the diagnosis of high-grade endometrial cancer. This might be categorized as overtreatment for early-stage POLE mutation patients. Due to a mixed carcinosarcoma component, the molecular detection post-tissue segmentation could not be performed. Thus, we were unable to determine whether LELC and ESC ensued from the same clonal origin.

## Conclusion

Future research should analyze more similar cases to elucidate the clinical characteristics, prognostic factors, and treatment responses associated with POLE mutations in endometrial cancers. Additionally, molecular pathology advancements might lead to novel targeted therapies and prognostic strategies, thereby enabling personalized treatment approaches. In summary, this case has enhanced the understanding of endometrial cancer research and can serve as an important reference for future clinical practice.

## Data Availability

The original contributions presented in the study are included in the article/Supplementary Material. Further inquiries can be directed to the corresponding authors.
